# Altered time structure of neuro-endocrine-immune system function in lung cancer patients

**DOI:** 10.1186/1471-2407-10-314

**Published:** 2010-06-21

**Authors:** Gianluigi Mazzoccoli, Gianluigi Vendemiale, Angelo De Cata, Stefano Carughi, Roberto Tarquini

**Affiliations:** 1 Unit of Internal Medicine and Chronobiology Unit, IRCCS Scientific Institute and Regional General Hospital, “Casa Sollievo della Sofferenza”, Opera di Padre Pio da Pietrelcina S.Giovanni Rotondo (FG), Italy; 2 Institute of Geriatrics, Department of Medical Science, University of Foggia, Italy; 3 Department of Internal Medicine, University of Florence, Florence (FI), Italy

## Abstract

**Background:**

The onset and the development of neoplastic disease may be influenced by many physiological, biological and immunological factors. The nervous, endocrine and immune system might act as an integrated unit to mantain body defense against this pathological process and reciprocal influences have been evidenced among hypothalamus, pituitary, thyroid, adrenal, pineal gland and immune system. In this study we evaluated differences among healthy subjects and subjects suffering from lung cancer in the 24-hour secretory profile of melatonin, cortisol, TRH, TSH, FT4, GH, IGF-1 and IL-2 and circadian variations of lymphocyte subpopulations.

**Methods:**

In ten healthy male volunteers (age range 45-66) and ten male patients with untreated non small cell lung cancer (age range 46-65) we measured melatonin, cortisol, TRH, TSH, FT4, GH, IGF-1 and IL-2 serum levels and percentages of lymphocyte subpopulations on blood samples collected every four hours for 24 hours. One-way ANOVA between the timepoints for each variable and each group was performed to look for a time-effect, the presence of circadian rhythmicity was evaluated, MESOR, amplitude and acrophase values, mean diurnal levels and mean nocturnal levels were compared.

**Results:**

A clear circadian rhythm was validated in the control group for hormone serum level and for lymphocyte subsets variation. Melatonin, TRH, TSH, GH, CD3, CD4, HLA-DR, CD20 and CD25 expressing cells presented circadian rhythmicity with acrophase during the night. Cortisol, CD8, CD8^bright^, CD8^dim^, CD16, TcRδ1 and δTcS1 presented circadian rhythmicity with acrophase in the morning/at noon. FT4, IGF-1 and IL-2 variation did not show circadian rhythmicity. In lung cancer patients cortisol, TRH, TSH and GH serum level and all the lymphocyte subsubsets variation (except for CD4) showed loss of circadian rhythmicity. MESOR of cortisol, TRH, GH, IL-2 and CD16 was increased, whereas MESOR of TSH, IGF-1, CD8, CD8^bright^, TcRδ1 and δTcS1 was decreased in cancer patients. The melatonin/cortisol mean nocturnal level ratio was decreased in cancer patients.

**Conclusion:**

The altered secretion and loss of circadian rhythmicity of many studied factors observed in the subjects suffering from neoplastic disease may be expression of gradual alteration of the integrated function of the neuro-immune-endocrine system

## Background

There are close relations between endocrine, nervous and immune system. Cortisol has a well recognized influence on immune function, inducing significant immunosuppression, characterized by the reduced cellular and humoral response of monocytes and B and T lymphocytes [1]. Melatonin, hormone secreted by the pineal gland, is able to influence the secretion of many endocrine glands, modulates the function of the immune system, shows antioxidative and oncostatic property and its production is under the control of the nervous system [2-5]. The pineal hormone directly stimulates activated helper T lymphocytes and plays an immunomodulatory role by opioid peptides [6] and by a thyroid-independent influence on the thymic function mediated by thyrotropin-releasing hormone (TRH) and thyroid-stimulating hormone (TSH) [7]. The insulin like growth factor (IGF)-1 is a potent mitogen for many cancer cell lines and the stimulation of IGF-1 receptor is necessary for proliferation of many cells in vivo and in vitro, often in conjunction with other growth factors [8]. Besides, recent studies have put in evidence that growth hormone (GH) and IGF-1 have an important role in stimulating lymphocyte production and function [9]. Abnormalities in hormone serum levels and in the proportions of various lymphocyte subpopulations have been found in a number of tumors [10]. The purpose of this study was to evaluate the 24-hour profiles of lymphocyte subsets and neuro-endocrine hormones with the aim to highlight alterations in patients suffering from lung cancer.

## Methods

The study was approved by the local Scientific and Ethical Committee (approval number MGRL001 approval date 6/4/2008). After obtainment of informed consent peripheral blood samples were collected at intervals of four hours for 24 hours starting at 06:00 h from ten healthy subjects (HS, mean age ± s.e. 57.2 ± 2.7, range 45-66) and from ten patients with untreated non small cell lung cancer (CP, mean age ± s.e. 56.6 ± 0.2, range 46-65). Inclusion criteria for healthy subjects were sex (male), age (<80 years), BMI (>25 and <30), normal physical activity level, no psychiatric disorder, no alcohol intake, no chronic conditions, normal blood pressure level. In all control subjects healthy status was assessed by medical history and physical examination, basal screening blood and urine test, ECG, chest X ray, upper and lower abdominal ultrasound scan. Inclusion criteria for subjects suffering from lung cancer were sex (male), age (<80 years), BMI (>25 and <30), normal physical activity level, performance status >80% by Karnofsky performance status scale or <2 by ECOG score, no psychiatric disorder, no alcohol intake, no chronic conditions, normal blood pressure level, tumor cell type (non small cell lung cancer, NSCLC) and the extent of the tumor was evaluated by clinical examination, bronchoscopy, computed tomography (CT) of the brain, chest, upper abdomen and ultrasonography of the liver. There were 3 cases of squamous cell carcinoma and 7 of adenocarcinoma. The pathological diagnosis was based on light microscopy according to the WHO classification. Tumors were staged according to the TNM classification of the International Union Against Cancer staging system after reviewing the clinical, radiologic, and pathologic data. The numbers of pT1, pT2, and pT3-4 cases were 3, 5, and 2 respectively. All 10 cases showed metastasis to regional lymph nodes. Regarding stage, the numbers of stage I, II, and III cases were 2, 5, and 3 respectively. The groups were matched closely to avoid sex, body mass index and seasonality of sampling related bias and all subjects were submitted to the same social routine, with identical mealtimes and sleep/wakefulness rhythm in the week preceding the sampling (lights on at 07:00 a.m. and lights out at 10:00 p.m., thereby supplying the subjects with 15 hours of light and 9 hours of darkness per day, 15:9 L:D). During the overnight sampling period a dim blue light (<100 lux) was used. In each blood sample we measured melatonin, cortisol, TRH, TSH, free thyroxine, GH, IGF-1 and IL-2. Blood was centrifuged immediately after collection and frozen at -20°C for later determination. All samples were analyzed in duplicate in a single assay; the intrassay and interassay coefficients of variation were below 13% and 16% respectively for melatonin, 10% and 9% for cortisol, 5% and 6% for TRH, 8% and 7% for TSH, 4% and 6% for FT_4_, 5% and 3% for GH, 3% and 8% for IGF-1, 5% and 7% for IL-2. Standard curves were run with every assay and the experimental values were derived from the curves. We measured melatonin by radioimmunoassay (Melatonin Radioimmunoassay Kit, Nichols Institute Diagnostics), cortisol by polarized light immuno-fluorescence assay (Cortisol TDx/TDxFLx, Abbott Laboratories), TRH by radioimmunoassay ("Frederic Joliot-Curie" National Research Institute for Radiobiology and Radiohygiene, Budapest, Hungary), TSH by immunoenzymatic assay (Enzymun-Test TSH, Boehringer Mannheim Immunodiagnostics), FT_4 _by immunoenzymatic assay (Enzymun-Test FT_4_, Boehringer Mannheim Immunodiagnostics), GH by immunoenzymometric assay (AIA-PACK HGH, Tosoh, Japan) and IGF-1 by radioisotopic assay (IGF-1 100T Kit, Nichols Institute Diagnostics), IL-2 by immunoenzymatic assay (IL-2 EIA, Technogenetics). In each blood sample we analyzed lymphocyte subpopulations (CD3, CD4, CD8, CD8 bright, CD8 dim, CD16, CD20, CD25, HLA-DR, TcRδ1, δTcS1) on peripheral blood anticoagulated with sodium ethylenediamine tetraacetic acid (EDTA). Analyses of lymphocyte subpopulations were performed on unfixed cell preparations with a fluorescence activated cell sorter (FACScan, Becton-Dickinson FACS Systems, Sunnyvale, California) and a panel of monoclonal antibodies (mAbs) to lymphocyte surface antigens (Ortho Diagnostic Systems: OKT3, OKT4, OKT8, OK-NK, OKB20, OKT26a, OK-DR; Thermo-Scientific, Rockford, IL, USA: TcRδ1 and δTcS1). mAbs were directly conjugated with phycoerythrin (PE) and to fluorescein isothiocyanate (FITC).  10 μl mAbs were added to 100 ml EDTA blood in Trucount tubes (BD Biosciences, San Jose, CA). After a 15-min incubation at room temperature the erythrocytes were disintegrated and after centrifugation the supernatants were washed with PBS. Non-lymphocytic cells contaminating the preparations were excluded from analysis using scatter gates set on the 90° light scatter profile. At least 10000 cells were acquired on the FACScan. Absolute counts of T cell subsets were calculated based on the proportion of the respective T cell subpopulation and on absolute counts obtained by the procedure. The number of fluorescent cells was expressed as a percentage of the total lymphocytes.

### Statistical analysis

Statistical evaluation of hormone serum levels and lymphocyte subpopulation values was performed by non-inferential descriptive biometric analyses, including one-way ANOVA performed between the timepoints for each variable and each group on original data and on data transformed as % of their individual 24 h mean to look for a time-effect, Student's *t *test and Mann-Whitney rank sum test, where appropriate, on MESOR, amplitude and acrophase values and on mean diurnal and nocturnal levels; a *p *value ≤ 0.05 was considered significant. The data were also analyzed by an inferential temporal descriptive biometric analysis using the methods named Single Cosinor and Population Mean Cosinor, based on a least-squares fit of a cosine curve to individual and grouped time series data, testing the occurrence of a 24 h rhythm (whether the zero-amplitude assumption is rejected at a probability level *p *≤ 0.05) and quantifying the parameters MESOR, Amplitude and Acrophase of the rhythm. MESOR is the acronym for Midline Estimating Statistic of Rhythm and defines the rhythm-determined average. Amplitude is the measure of one half the extent of rhythmic change in a cycle estimated by the function used to approximate the rhythm. Acrophase, measure of timing, is the phase angle of the crest time in the function appropriately approximating a rhythm, in relation to the specified reference timepoint. Chronobiologic analysis was performed with Cosinor 2.2. ANOVA, Student's *t *test and Mann-Whitney rank sum test were performed with SigmaPlot11.0 [11].

## Results

**Figure 1:**
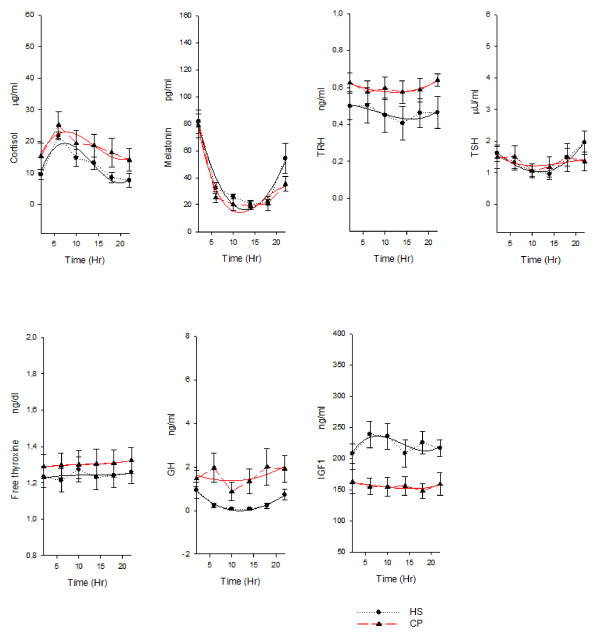
**Nyctohemeral variation of hormone serum levels**. *x-y *plot showing 24-hour time qualified profiles of cortisol, melatonin, TRH, TSH, free thyroxine, GH and IGF 1 expressed as mean ± s.e. calculated on single time point values from ten healthy subjects and ten patients suffering from non small cell lung cancer. Cubic regression function data interpolation represented as best fit solid line superimposed on the raw data (dotted line).

**Figure 2:**
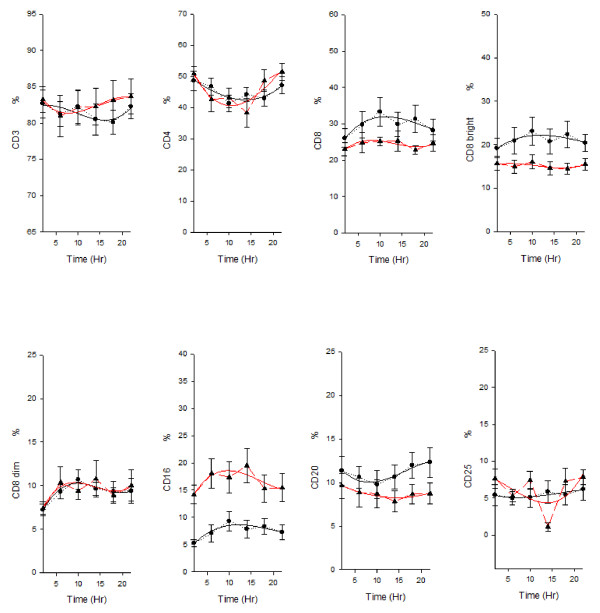
**Nyctohemeral variation of lymphocyte subpopulations**. *x-y *plot showing 24-hour time qualified profiles of lymphocyte subset percentages (CD3, CD4, CD8, CD8^bright^, CD8^dim^, CD16, CD20, CD25) expressed as mean ± s.e. calculated on single time point values from ten healthy subjects and ten patients suffering from non small cell lung cancer. Cubic regression function data interpolation represented as best fit solid line superimposed on the raw data (dotted line).

**Figure 3:**
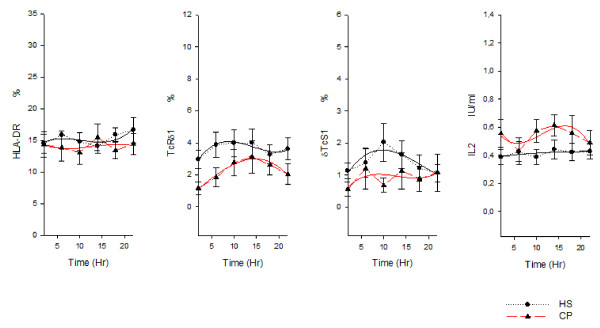
**Nyctohemeral variation of lymphocyte subpopulations**. *x-y *plot showing 24-hour time qualified profiles of lymphocyte subset percentages (HLA-DR, TcRδ1, δTcS1) and IL-2 serum levels expressed as mean ± s.e. calculated on single time point values from ten healthy subjects and ten patients suffering from non small cell lung cancer. Cubic regression function data interpolation represented as best fit solid line superimposed on the raw data (dotted line).

In the healthy subjects ANOVA showed a time effect for cortisol, melatonin, TRH, TSH, GH, CD3, CD4, CD8, CD8^bright ^, CD8^dim ^, CD16, TcRδ1 and δTcS1 and a clear circadian rhythm was validated for the variation of hormone serum levels and of lymphocyte subset percentages: melatonin, TRH, TSH, GH, CD3 (total T cells), CD4 (T helper/inducer subset), HLA-DR (B cells and activated T cells), CD20 (total B cells) and CD25 (T activated lymphocytes with expression of the α chain of IL-2 receptor) presented circadian rhythmicity with nocturnal acrophase. Cortisol, CD8 (T suppressor/cytotoxic subset), CD8^bright ^(T suppressor subset), CD8^dim ^(T cytotoxic subset), CD16 (natural killer cells), TcRδ1 (epitope of the constant domain of δ chain of TCR) and δTcS1 (epitope of the variable domain of δ chain of TCR) presented circadian rhythmicity with diurnal acrophase. FT4, IGF-1 and IL-2 variation did not show circadian rhythmicity. In lung cancer patients ANOVA showed a time effect for cortisol, melatonin, TSH, CD4, CD16, CD25, TcRδ1 and IL2. Cortisol, TRH, TSH and GH serum level and all the lymphocyte subsubsets variation (except for CD4) showed loss of circadian rhythmicity. MESOR of cortisol, TRH, GH, IL-2 and CD16 was increased in cancer patients, whereas MESOR of TSH, IGF-1, CD8, CD8^bright^, TcRδ1 and δTcS1 was decreased in cancer patients (Figure 1, figure 2 and figure 3).

When we compared mean diurnal levels (mean of 06:00h-10:00h-14:00h) GH (p < 0.001) and CD16 (p < 0.01) levels were higher in cancer patients, whereas IGF1 (p < 0.001), CD8 (p = 0.03) and CD8^bright ^(p = 0.001) levels were lower in cancer patients. When we compared mean nocturnal levels (mean of 18:00h-22:00h-02:00h) cortisol (p = 0.02), TRH (p = 0.03), GH (p < 0.01), CD16 (p < 0.0001) and CD25 (p = 0.03) levels were higher in cancer patients, whereas melatonin (p = 0.04), TSH (p = 0.02), IGF-1 (p < 0.0001), CD8 (p = 0.01), CD8^bright ^(p = 0.001), CD20 (p = 0.03), TcRδ1 (p = 0.001) and δTcS1 (p = 0.02) levels were lower in cancer patients. The melatonin/cortisol mean nocturnal level ratio was decreased in cancer patients (*p *= 0.01)

Table 1 shows chronobiological data derived from best fitting sine curves (fitted period:24 hours = 360°), F and p value from ANOVA performed between the timepoints for each variable and p value from Student's t test and Mann-Whitney rank sum test, where appropriate, on MESOR, amplitude and acrophase values between healthy subjects and lung cancer patients. Figure 1 and 2 show *x-y *plot depicting 24-hour time qualified profiles of lymphocyte subset percentages and hormone serum levels.

**Table 1:** Chronobiological data derived from best fitting sine curves (fitted period:24 hours = 360°) and *F *and *p *value from ANOVA performed between the timepoints for each variable

Healthy subjects										Lung cancer patients						
Factor	*p#*	MESOR ± SE	Amplitude ± SE	Acrophase ± SE	ANOVA				*p#*	MESOR ± SE	Amplitude ± SE	Acrophase ± SE	ANOVA			
					Original Units		% of mean						Original Units		% of mean	
					*F*	*p*	*F*	*p*					*F*	*p*	*F*	*p*
Cortisol	0.027	12.52 ± 1.53	5.85 ± 1.97	08:08 ± 01:18	6.3	0.01	22.3	<0.01	0.138	17.85 ± 1.03*	4.21 ± 1.39	09:00 ± 01:32	1.1	0.41	5.1	<0.01
Melatonin	0.013	38.75 ± 5.43	26.24 ± 7.32	01:13 ± 01:15	14.2	<0.01	25.4	<0.01	0.036	33.12 ± 7.34	23.12 ± 9.94	01:28 ± 01:37	4.9	<0.01	12.3	<0.01
TRH	0.024	0.45 ± 0.09	0.04 ± 0.05	02:47 ± 01:05	0.1	0.97	4.5	0.01	0.245	0.60 ± 0.02*	0.03 ± 0.04	23:41 ± 01:48	0.3	0.35	0.5	0.46
TSH	0.020	1.38 ± 0.04	0.45 ± 0.03	23:12 ± 00:32	2.3	0.08	12.5	0.01	0.328	1.33 ± 0.09*	0.18 ± 0.08	00:05 ± 02:05	0.6	0.78	3.9	0.01
FT4	0.897	1.24 ± 0.06	0.01 ± 0.03	15:52 ± 08:08	0.3	0.95	1.4	0.15	0.377	1.33 ± 0.02	0.02 ± 0.03	18:44 ± 02:13	0.4	0.96	0.8	0.72
GH	0.039	0.38 ± 0.03	0.45 ± 0.02	00:22 ± 00:50	3.9	0.01	5.8	<0.01	0.447	1.55 ± 0.17*	0.38 ± 0.23	22:16 ± 02:36	0.5	0.61	1.3	0.23
IGF-1	0.578	221.01 ± 6.0	9.34 ± 8.12	08:28 ± 03:24	0.2	0.76	3.4	0.09	0.424	156.02 ± 1.88*	4.05 ± 2.36	02:41 ± 02:26	0.1	0.89	1.7	0.29
CD3	0.014	82.10 ± 0.31	0.98 ± 0.53	03:17 ± 22:09	0.3	0.96	2.3	0.04	0.135	83.01 ± 0.21	1.08 ± 0.25	20:49 ± 01:21	0.7	0.76	1.9	0.21
CD4	0.033	45.12 ± 0.75	3.11 ± 1.02	01:16 ± 01:17	1.5	0.36	4.0	<0.01	0.035	43.83 ± 1.27	6.13 ± 1.47	22:48 ± 00:53	2.3	0.04	6.2	0.01
CD8	0.024	29.61 ± 0.74	2.59 ± 1.04	12:48 ± 01:37	0.4	0.76	4.2	0.01	0.531	23.12 ± 0.36*	0.52 ± 0.46	11:21 ± 03:04	0.4	0.98	0.9	0.61
CD8 Bright	0.039	21.06 ± 0.54	1.35 ± 072	13:34 ± 02:04	0.6	0.84	1.4	0.04	0.597	15.21 ± 0.26*	0.42 ± 0.36	04:45 ± 03:19	0.3	0.93	0.7	0.72
CD8 Dim	0.028	9.22 ± 0.39	1.10 ± 0.49	12:37 ± 01:58	1.5	0.35	3.7	0.01	0.631	9.45 ± 0.43	0.82 ± 0.69	12:46 ± 03:40	0.6	0.62	1.2	0.39
CD16	0.015	7.89 ± 0.21	1.42 ± 0.36	13:24 ± 01:37	0.7	0.51	2.8	0.01	0.187	16.33 ± 0.41*	2.12 ± 0.37	11:16 ± 01:32	0.5	0.81	2.3	0.05
CD20	0.006	11.21 ± 0.04	1.21 ± 0.08	21:24 ± 00:21	0.4	0.98	2.3	0.09	0.129	8.73 ± 0.12	0.61 ± 0.45	02:21 ± 01:16	0.3	0.89	1.0	0.56
CD25	0.040	5.52 ± 0.12	0.44 ± 0.19	19:46 ± 01:34	0.2	0.93	2.4	0.08	0.526	7.02 ± 0.31	0.68 ± 0.32	20:12 ± 03:04	0.6	0.80	3.9	<0.01
HLA-DR	0.033	15.28 ± 0.39	0.69 ± 0.56	22:33 ± 03:21	0.6	0.67	2.2	0.07	0.893	14.02 ± 0.41	0.28 ± 0.49	18:47 ± 08:06	0.1	0.54	0.5	0.83
TcR δ 1	0.026	3.59 ± 0.11	0.41 ± 0.18	11:12 ± 01:33	0.2	0.89	3.2	0.04	0.082	2.24 ± 0.02*	0.94 ± 0.15	14:09 ± 00:22	1.2	0.39	3.6	0.01
δTcS1	0.036	1.43 ± 0.05	0.43 ± 0.06	10:52 ± 00:44	0.4	0.76	2.9	0.03	0.918	0.91 ± 0.20*	0.07 ± 0.18	14:56 ± 09:32	0.2	0.78	2.1.	0.08
IL-2	0.424	0.43 ± 0.02	0.05 ± 0.03	14:53 ± 02:32	0.2	0.98	0.3	0.94	0.627	0.52 ± 0.03*	0.02 ± 0.04	17:01 ± 03:10	0.4	0.86	1.0	0.05

Units: μg/dl for cortisol, pg/ml for melatonin, ng/ml for TRH, μU/ml for TSH, ng/dl for FT_4_, ng/ml for GH, ng/ml for IGF-I, percentage for lymphocyte subpopulations, IU/ml for IL-2; all parameters analyzed in all the subjects; Hr:Mn for acrophase; all parameters analyzed in all the subjects. *# p *value from an *F*-test of the null amplitude rejection hypothesis (for a rhythm with a chosen period *τ*); * *p *value < 0.05 from *t*-test or Mann-Whitney rank sum test as indicated performed on MESOR, amplitude and acrophase values

## Discussion and Conclusion

Body homeostasis is maintained by a perfect integration among the nervous, endocrine and immune system involving different glands and organs and guided by a spatio-temporal sequence of tissue-specific events, hormone and cyto-chemokine/receptor interaction and modulation processes. The pineal gland is innervated by post-ganglionic nervous fibers coming from the superior cervical ganglion, that receives fibers from the suprachiasmatic nucleus of hypotalamus, innervated by the retinohypothalamic tract. Changes in lighting condition influence activity of the retino-hypothalamic-pineal system, with inhibiting effect on melatonin production, controlled also by an endogenous free-running pacemaker located in the suprachiasmatic nucleus [12]. The melatonin mediated photoperiodic message is a fundamental cue for the circadian and seasonal coordination of biological functions. Melatonin plays a role of immunomodulation by opiatergic ways and stimulates activated helper T lymphocytes to produce opioid agonists and cytokines (IL-2 and IL-4) [13,14]. The immunomodulatory role of melatonin may also be exerted by an influence on the thymic function mediated by TRH and TSH, able to counteract in experimental conditions thymic involution induced by prednisolone (this effect seems to be thyroid-independent and not correlated to thyroxine levels) [15].

TRH and TSH secretion changes with circadian periodicity and nightly peak hormone serum levels have been described. Thyroxine (T_4_) and triiodothyronine (T_3_) serum levels present minor changes during a 24-h period, their fluctuations are shortlived and diurnal variations in hormone levels can be demonstrated in most, but not in all instances. In our control subjects TRH and TSH blood levels show circadian variations with higher levels during the night, whereas thyroid hormone levels do not change with circadian rhythmicity. Melatonin may regulate the response of thyroid to hypothalamic-pituitary axis stimulation, playing a modulatory action on hypothalamic-pituitary-thyroid axis and functioning as a servo-mechanism that diminishes or increases responses when stimuli are respectively too strong or too low [16,17]. This phenomenon, called feed-sideward, takes part also in the interaction among the pineal gland and the hypothalamic-pituitary-adrenal axis [18].

GH and IGF-1 have been recognized as stimulators of lymphopoiesis and immune function. IGF-1 is one of the most important growth factors for normal cell proliferation and several tumor cell lines have recently appeared to be also stimulated by this mitogen: it acts as an endocrine hormone via the blood and as paracrine and autocrine growth factor locally. GH stimulates the biosynthesis of IGF-1 in liver and in other organs and tissues and an autocrine or paracrine GH/IGF-1 system have been evidenced in lymphoid tissues, capable of influencing lymphopoiesis and immune function [19-21].

Neoplastic diseases are characterized by an altered pattern of hormonal secretion, with changes that may be qualitative (loss of circadian rhythmicity) and/or quantitative (increase or decrease of serum levels) as variously described by many scientific studies [22-25].

Immune responses show temporal variations, related to circadian changes of total lymphocytes and specific lymphocyte subsets in the peripheral blood, of antibodies and cell mediated immune responses, with an inverse relationship to plasma cortisol concentration [26-30]. Cortisol has a well recognized influence on immune function, inducing significant immunosuppression, characterized by the reduced cellular and humoral response of monocytes and B and T lymphocytes [31]. There are different circadian variations of the total number of circulating immune cells and of specific lymphocyte subpopulations [32,33]. As evidenced in our healthy control subjects, circulating T and B cell percentages levels change with circadian rhythmicity in antiphase with the rhythm of cortisol and this rhythm of variation is recognizable for the changes of total T cells, T helper/inducer subset, B cells and activated T cells, total B cells, T activated lymphocytes with expression of the α chain of IL-2 receptor. The levels of T suppressor/cytotoxic lymphocytes, natural killer cells and γδTCR expressing cells are higher in the morning. Immune system plays an important role in the defense against neoplastic disease: there exists an effective T-cell mediated immune surveillance capable of monitoring the genetic integrity of mammalian cells, T lymphocytes are an essential component of specific immune responses wich produce tumour rejection and endocrine and immune alterations have been evidenced in cancer patients.

Data obtained in our study shows that in lung cancer patients cortisol, TRH, TSH and GH secretion is altered with loss of circadian rhythmicity. The disappearance of circadian rhythm of cortisol secretion is typical of hypercortisolism and adding to the reduced nocturnal melatonin secretion determines in our cancer patients the observed decrease of the melatonin/cortisol ratio. An altered pattern of cortisol secretion (absence of circadian rhythmicity and increased production), together with reduced nightly melatonin secretion, may represent a marker of neuro-endocrine system disorder [34]. The loss of circadian rhtyhmicity of cortisol serum level variation may be caused by an alteration of central nervous system (CNS) regulatory mechanisms of cortisol secretion and may be involved in a dual relationship to the melatonin secretion pattern [35]. The pineal gland influences the function of the hypothalamus-hypophysis-adrenal axis, melatonin regulates glucocorticoid receptors and adrenal corticosteroids modulate pineal melatonin synthesis [36]. Data obtained in previous studies indicate that the pineal function is significantly impaired in patients with altered pattern of cortisol secretion or after the administration of dexamethasone [37,38]. The melatonin/cortisol ratio, calculated on the mean of nocturnal serum levels or on overnight urinary cortisol and melatonin (or its metabolite 6-hydroxy-melatonin-sulphate), may be a valuable and reliable method for population and individual screening for cancer at scheduled time intervals (for example, at yearly interval in the general population and at half-yearly interval or quarterly in subjects at elevated risk for neoplastic disease). There are reciprocal and different influences also among melatonin and male and female gonadal steroids and this is the reason why we have considered only male subjects to avoid sex related biases.

In our cancer patients GH serum levels are increased, but the nyctohemeral pattern of secretion is lost and this determines an unbalanced relationship between GH secretion and IGF-1 serum levels, that are decreased. Studies conducted on GH-deficient patients have demostrated that different time treatment schedules of GH administration have different effects on IGF-1 serum levels and the closest similarity to normal hormone and metabolite patterns and relationships is reached by GH injection in the evening [39]. All the patients enrolled in our study had normal BMI, so that the low levels of serum IGF-1 found in cancer patients should not be related to the state of nutrition. A global disorder of the neuro-endocrine axes has to be considered and may also explain the increase of TRH levels and the decrease of TSH levels observed in our cancer patients. An important causal factor may be represented by the altered time structure of GH and TRH secretion evidenced in our lung cancer patients, but another explanation may be represented by hormone resistance. IL-2 serum levels are increased in our cancer patients, maybe following immune activation and maybe representative of a global increase of the others cyto/chemokines that mediate inflammation [40]. The changes in the TRH/TSH axis and GH/IGF-1 axis function in the patients suffering from cancer may be mediated by the inflammatory cytokines. Evidence from cell culture and animal experiments suggests that interleukin-1 (IL-1) and tumour necrosis factor-α (TNFα) can cause GH resistance. Increased circulating levels of the inflammatory cytokines and/or increased *in vitro *production by peripheral blood mononuclear cells have been reported in cancer patients. GH resistance with respect to the stimulation of hepatic IGF1 production has been observed in the lung cancer patients and GH resistance has been associated with an acute-phase response. The effects of the inflammatory cytokines are likely to be mediated by complex paracrine pathways, rather than 'endocrine' pathways [41,42].

The neuro-endocrine-immune system function is characterized by a complex time structure composed of multiple rhythms in different frequency ranges. The rhythms of the same frequency may have the same phase or different phases and usually show a well defined time-relation to each other. The loss of the array of rhythms or a change of their functional interactions may alter the organism's time structure leading to chronodisruption and internal desynchronization. The alteration of the organism's time structure may lead to functional disturbances and may impair repairing and defensive mechanisms. Cancer may arise from chronodisruption and may worsen internal desynchronization. A complete loss of rhythmicity or a change of phase of the rhythms are the most frequent alterations, but another important factor is represented by the change of levels and amplitude of variation.

The multifrequency structrure that characterizes the function of the immune system and the complexity of the time qualified variations of its different components has to be taken in consideration when we approach functional evaluations, clinical interpretations and therapeutical interventions. Among the lymphocyte subsets considered in our study, the CD8 bearing cells and in particular the T suppressor subset are diminished in lung cancer patients [43,44]. The decrease of T suppressor subpopulation has been observed in other types of cancer (gastric cancer, colorectal carcinoma and urological cancer) [45-47] and may represent a marker of immunological disorder. As evidenced in our study, the natural killer cells are increased in cancer patients, whereas the expression of the constant and variable domains of δ chain of T-cell receptor 1 is decreased and this may be an important finding as γδTCR complex is mainly expressed at the cell surface of cytotoxic lymphocytes, is involved in T cell activation and activated γδ expressing cells frequently exhibit cytotoxic activity against multiple target cell lines including neoplastic cells [48].

The altered time structure and serum level evidenced in our study for hypothalamic (TRH), pituitary (GH, TSH), pineal (melatonin) and adrenal (cortisol) secretion may be produced by and/or may increase the altered interaction among nervous, endocrine and immune system. Lung cancer patients show loss of circadian rhythmicity of all lymphocyte subpopulations studied except for CD4. This phenomenon may impair an efficient immune response, may be related to altered hormone serum levels and to changes of the natural circadian rhythmicity and may cause the loss of the physiological timed windows of interaction among neural/endocrine structures and immune effectors, considering also that immune cells have hormone binding sites and hormone production capability. The reduction in number of CD8 and γδTCR expressing cells in the peripheral blood may represent a marker of immunological disorder or may follow homing in pathological and neoplastic tissue as effector cells and/or regulatory cells.

In conclusion, our results have indicated that lung cancer is associated with disordered hormonal secretion and anomalies of proportion and circadian variation of lymphocyte subsets, probably expression of impaired integration among nervous, endocrine and immune system in front of advancing neoplastic disease. These systems show natural patterns of circadian rhythmicity and it is important to understand how multiple rhythms relate to each other to maintain an internal order and how the loss of this internal order may contribute to the beginning and the progression of neoplasticdisease.

## Competing interests

'The authors declare that they have no competing interests'.

All the Authors have read and approved the submission of the present version of the manuscript and that the manuscript has not published and is not being considered for publication elsewhere in whole or in part in any language except as an abstract.

In the past five years we have not received reimbursements, fees, funding, or salary from an organization that may in any way gain or lose financially from the publication of this manuscript, either now or in the future. No organization is financing this manuscript (including the article-processing charge).

We do not hold any stocks or shares in an organization that may in any way gain or lose financially from the publication of this manuscript, either now or in the future

We Do no hold or are currently applying for any patents relating to the content of the manuscript. We have not received reimbursements, fees, funding, or salary from an organization that holds or has applied for patents relating to the content of the manuscript.

We have no other financial competing interests. There are no non-financial competing interests (political, personal, religious, ideological, academic, intellectual, commercial or any other) to declare in relation to this manuscript.

## Authors' contributions

All authors have read and approved the final manuscript. GM: conception and design of the study, data collection, analysis and interpretation of data, carried out statistical analysis and the draft of the manuscript. GV: critical revisal of the manuscript. AD: interpretation of data, carried out part of the draft of the manuscript. SC: data collection, data interpretation, carried out part of the draft of the manuscript. RT: critical revisal of the manuscript, interpretation of data.

## Pre-publication history

The pre-publication history for this paper can be accessed here:

http://www.biomedcentral.com/1471-2407/10/314/prepub

## Aknowledgments

We are greatly indebted to Professor Franz Halberg and we wish to express our gratitude to him for introducing us to the study of Chronobiology
